# Voluntary and spontaneous facial mimicry toward other’s emotional expression in patients with Parkinson’s disease

**DOI:** 10.1371/journal.pone.0214957

**Published:** 2019-04-11

**Authors:** June Kang, Dilara Derva, Do-Young Kwon, Christian Wallraven

**Affiliations:** 1 Korea University, Department of Brain and Cognitive Engineering, Seoul, South Korea; 2 Empathy Research Institute, Seoul, South Korea; 3 Korea University Ansan hospital, Department of Neurology, Ansan City, South Korea; University of Toronto, CANADA

## Abstract

A “masked face”, that is, decreased facial expression is considered as one of the cardinal symptoms among individuals with Parkinson’s disease (PD). Both spontaneous and voluntary mimicry toward others’ emotional expressions is essential for both social communication and emotional sharing with others. Despite many studies showing impairments in facial movements in PD in general, it is still unclear whether voluntary, spontaneous, or both types of mimicry are affected and how the impairments affect the patients’ quality of life. We investigated to verify whether impairments in facial movements happen for spontaneous as well as for voluntary expressions by quantitatively comparing muscle activations using surface electromyography. Dynamic facial expressions of Neutral, Anger, Joy, and Sad were presented during recordings in corrugator and zygomatic areas. In the spontaneous condition, participants were instructed to simply watch clips, whereas in the voluntary condition they were instructed to actively mimic the stimuli. We found that PD patients showed decreased mimicry in both spontaneous and voluntary conditions compared to a matched control group, although movement patterns in each emotion were similar in the two groups. Moreover, whereas the decrease in mimicry correlated with the decrease not in a health-related quality of life index (PDQ), it did so in a more subjective measurement of general quality of life index (SWB). The correlation between facial mimicry and subjective well-being index suggests that the ‘masked face’ symptom deteriorates patients’ quality of life in a complex way affecting social and psychological aspects, which in turn may be linked to the increased depression risk among individuals with PD.

## Introduction

As Darwin (1872) highlighted, facial expressions play an essential role in emotional communication. People not only express their own emotion through facial expressions, but also understand and share other peoples’ emotions by mimicking their facial expression through facial feedback mechanisms [1, 2].

Facial mimicry of emotional expressions is a hard-wired mechanism that is shared with other primates [3] and is observed from early infanthood [4]. Psychophysiological studies using facial electromyography (EMG) have indicated that the facial mimicry is preserved in adults [7,8], and elderlies [5, 6]. Through adolescence, visible mimicry decreases, but its continued presence can be identified using muscle-based measurements methods like surface EMG.

Both voluntary and spontaneous mimicry have significance in social life. They help to understand observed emotional expressions of others [7], promote the empathic process [2], and facilitate social bonding [8]. Blocking spontaneous facial mimicry hampers emotion recognition [1, 9] as does the suppression of voluntary mimicry [10]. Facial mimicry also serves an affiliative function, promoting interpersonal relationship [8], and affectionate responses by the person who is being mimicked towards the mimicker and vice versa [11].

Parkinson’s disease (PD) is the second most common neurodegenerative disease and is caused by the loss of dopaminergic neurons in the substantia nigra. Clinicians have reported decreased facial expression or 'masked face' as one of the cardinal symptoms of PD [12]. Despite its frequent occurrence in clinical literature [13–15], only a few studies so far have investigated alteration of facial mimicry quantitatively [16, 17], and little is known about its effect on the quality of life of patients with PD. Since facial expressions are an important aspect of social life, a deeper understanding of this symptom is increasingly important, when considering both the increasing life expectancy of PD patients on the one hand and the elevated risk of depression on the other hand [18].

With respect to the effects of the mask-like face in PD, results are unclear as to whether it impairs only spontaneous expressions or voluntary ones or both. Historically, the symptom has been intepreted as diminished *spontaneous* facial expressions, since most of the studies focused on only spontaneous expressions. Studies found that PD patients were impaired in spontaneous emotional expressions during conversation [19], and in response to emotional evocative stimuli [19, 20]. On the other hand, several studies have also reported that PD patients have difficulty in *voluntary* facial expressions [19, 21].

For spontaneous expressions, Katsikitis and Pilowsky [20] presented a series of cartoons and when comparing the smiling behavior of PD patients to a control group found that both frequency and strength of smiling was reduced. Using the same paradigm, they compared PD patients to people with major depressive disorders (MDD), again with PD patients showing diminished frequency responses and strength of zygomaticus response similar to MDD group [22]. Based on the comparison of recorded semi-structured interviews of four PD and four control subjects, Pitcairn and colleagues reported fewer spontaneous smiles in the PD group. These authors also argued qualitatively that the smiles of PD patients seemed less genuine compared to those of the controls [23]. Another study directly measured the spontaneous muscle response using surface electromyography without explicit instruction to mimic [16]: although spontaneous mimicry in both corrugator regions toward negative emotions and in the zygomatic region toward positive emotion were preserved to a certain extent, PD patients showed reduced and delayed muscle activation.

In the context of voluntary expressions, reduced emotional facial expression in response to verbal commands were reported in a few studies. In a more structured approach, Smith et al. [24], adopted the Facial Action Coding System to analyze both spontaneous and voluntary facial expressions and found that PD patients displayed less facial emotional reaction to emotionally-laden film clips compared to controls in *both* conditions. Bowers and colleagues used computer vision techniques to quantitatively analyze videos of facial expressions and concluded that PD patients show less and slower voluntary facial movements [25]. Using a 3D optoelectronic system, Bolonga and colleagues assessed voluntarily mimicked facial expressions of emotion [26]. They found a slower velocity and lower amplitude in the PD group compared to controls while mimicking basic emotions. Significantly lower amplitudes were observed in Anger, Disgust, Happiness, and Sadness, but slowed peak-velocities were observed in all six emotions. A similar result has been reported in a study with an observer rating approach to quantifying the facial expressions [17]. Here, PD patients and controls were asked to pose facial expressions of six basic emotions, and video clips were recorded. Then independent, healthy raters responded to images of the facial expression selected from the recorded video with both emotion recognition tasks and Likert-style amplitude assessments. The raters recognize the facial expressions of the control group significantly better compared to the ones of the PD group. For the three emotions of Happiness, Anger, and Sad, recognizability as well as perceived expressivity and confidence levels were significantly lower, and raters’ response time was also significantly longer in the recognition task.

Together, the findings from previous studies support impairment in both spontaneous and voluntary facial expressions of emotion in PD patients. Only a few investigations so far, however, directly measured the alteration of both voluntary and spontaneous mimicry in a quantitative way [16, 17]. The present study adopted such a quantative approach, testing both voluntary and spontaneous mimicry using a within-subject design while controlling for potential order effects. Importantly, recent work has shown that dynamic facial expressions are not only more ecologically valid stimuli [27] but also elicit stronger muscle activation compared to static ones [28, 29]. Since previous studies largely relied on static image stimuli, the present study adopted FACS-based, dynamic facial stimuli. Lastly, here we also focused on how facial mimicry and subjective well-being of PD patients may be connected. The masked face caused by PD might also lower the quality of life of PD patients [30, 31], however, no study investigated this connection directly so far. Here, this relationship was probed not only a quality-of life measurement in a disease context (PDQ), but also with an index reflecting more general and psychological aspects of quality of life (SWB).

In summary, the study hypothesizes that 1) individuals with PD will show a lower amplitude of facial mimicry in both voluntary and spontaneous mimicry conditions, and that 2) the level of facial mimicry will be correlated with quality of life measures in PD patients.

## Materials and methods

### Participants

This study recruited patients who visited the movement disorder clinic of a university-affiliated hospital and were diagnosed with PD according to the clinical diagnostic criteria of the United Kingdom Parkinson's Disease Society Brain Bank. All participants were provided with informed consent which they all signed. They underwent thorough neurological examinations by an experienced neurologist specialized in movement disorders.

Participants diagnosed with depression based on criteria from the fourth edition of the Diagnostic and Statistical Manual of Mental Disorders (DSM-IV-TR) for major depressive disorder (MDD) were excluded as severe depression might affect the results of the examinations. Patients with a K-Mini-Mental State Examination (K-MMSE) score less than 24, or compatible to the diagnosis of early dementia on full neuropsychological tests, as well as either previous focal or diffuse brain lesions in brain MRI were also excluded. PD patients with a previous history of stroke, other neurological or psychiatric disorders, a history of medication that might cause parkinsonism, or secondary causes of dementia were also excluded.

All of the PD participants were asked to withdraw from their dopaminergic drugs at least 12 hours before the study. The age-matched control group was recruited from a local senior community through advertisements. The inclusion criteria for the control group were seniors over 50 years old without any previous psychiatric or neurological symptoms.

### Ethics statement

This study was approved by the Research Ethics Committee involving Human Beings of the

Korea University Ansan hospital (protocol: K-2373). All guardians responsible for the patients and controls signed a free and informed consent form.

#### Mental Well-Being scale (MWB)

The MWB is a scale used to measure the mental well-being based on the theory of Keyes [32], who suggested that there are three aspects of happiness: emotional well-being (EWB, e.g., happiness or satisfaction), psychological well-being (PWB, e.g., personal growth), and social well-being (SWB, e.g., social integration). Each item was rated on a 6-point Likert-type scale. The resulting total score ranged from 0 to 70. The Korean, validated version of the MWB [33](Lim et al., 2013) was used here in this study [34].

#### Health-related quality of life

The Korean version of 39-item Parkinson’s disease questionnaire (PDQ-39) was used to assess the health related quality of life especially focused on PD patients [35].

#### Symptom severity and alexithymia

The clinical severity of PD group was measured by the Unified Parkinson's Disease Rating Scale (UPDRS) Part III [36]. The Korean version of the 20-item Toronto Alexithymia Scale (TAS-20K) was used to assess alexithymia symptoms [37].

#### Apparatus

Stimuli were presented on a 15inch laptop (Macbook Pro, Apple, Inc, CA, USA) at 60Hz with SuperLab software (version 4.5, Cedrus, Inc., AZ, USA). All psychophysiological measurements were performed using MP150 data acquisition system (Biopac Systems, Inc., CA, USA) with the Acknowledge software (version 4.1) running on an OS X system. Synchronization between the stimulus presentation system and the physiological data acquisition system was achieved by the StimTracker system (Cedrus, Inc., AZ, USA).

#### Stimulus

This study employed dynamic facial expressions rather than static ones as stimuli since they are ecologically more valid: dynamicity is an intrinsic property of facial expressions, affecting behavioral [32], psychophysiological [22], and neural activity [23] aspects. Anger, Joy, Sad, and Neutral expressions were selected, since those expressions are known to initiate vivid and discriminable muscle activity in Corrugator and Zygomatic regions [8] and since a previous study reported that people are significantly worse at recognizing these three emotions in PD patients [17]. Expression stimuli were chosen from the East-Asian Dynamic Facial Expression Stimulus (EADFES) database [33], which contains stimuli created and verified based on suggested action units related to each emotion (Anger: 4CDE+5CED+7+17+23/24, Joy: 6+12CDE+25, Sad: 1+4+15ABC+17, [38]). Average recognition accuracy in a forced-choice task for the selected expressions was 86.90%. On average, each expression was 2000ms long.

### Procedure

#### Facial EMG measurement

The study selected regions in the face related to the corrugator muscle (AU4) and the zygomaticus major muscle (AU12). The strength of each muscle response to the facial stimuli was recorded with 4mm Ag/AgCl cup electrodes, and amplified bi-polarly using an EMG100C preamplifier (1-500Hz bandpass filter, 60Hz notch filter, and gain factor of 2000), and digitized at 2000Hz with a MP150 system (Biopac Systems, Inc., CA, USA). The digitized EMG signal was integrated and smoothed in a 100ms time window.

#### Experimental procedure

The participants were tested individually in a quiet room. Each participant conducted a sequence of 80 trials, in which a video was first shown followed by passively observation (spontaneous condition) or active mimicry (voluntary condition). The sequence of voluntary and spontaneous mimicry conditions was pseudo-randomized for every participant. Similarly, the stimulus presentation followed a pseudo-random schedule.

### Data reduction and statistical analysis

#### Data reduction

EMG results of the mimicry trials were averaged in 15 non-overlapping intervals of 200ms each, starting 500ms after stimulus onset. Statistical analyses were then performed on different scores with the averaged facial EMG response of 500ms before stimuli presentation subtracted from the averaged facial EMG responses to each facial expression in each of the intervals.

#### Statistical analysis

Fixed effects, random effects and mixed-effects models of variables to account for the repeated measurements of participants over time, were run using SAS PROC Mixed, with an unstructured covariance structure. The REML (Restricted maximum likelihood) method was adopted since it provides more accurate results when sample size (especially the number of higher-level units) is small. A multivariate analysis was performed on the EMG scores with intervals (15×200ms intervals) and emotion (neutral, anger, joy, sad) as within-subject factors, and group (PD vs. controls) as a between-subject factor. Effect intensities were evaluated using Cohen's f^2^ and interpreted as small (f^2^ ≥ 0.02), medium (f^2^ ≥ 0.15), or large effects (f^2^ ≥ 0.35). A PEARSON correlation analysis was conducted between mean and maximum integrated EMG scores with subjective well-being score, alexithymia trait score, and PDQ scores.

## Results

### Demographics

This study included 20 PD patients (male: 9, female: 11, mean age = 58.50 (SD 8.64), mean education years = 11.25 (SD = 4.60)) and 20 age-matched healthy controls participated (male: 7, female: 13, mean age = 59.00 (SD 6.88), mean education years = 12.50 (SD = 3.14)). There were no significant differences between the two groups with respect to age, sex, education years and K-MMSE. The PD patients’ Alexithymia score (TAS-20) (t = .318, p = 0.753) and emotional expressivity score (EES) (t = 1.453, p = 0.154) were also not significantly different from those of the controls. In contrast, the PD group had significantly lower subjective scores in well-being (t = 4.152 p < .001), psychological well-being (t = 4.489, p < .001), and social well-being (t = 4.293, p < .001) compared to controls. The difference in the emotional well-being score was not significant (t = 1.953, p = .058).

#### Voluntary mimicry condition

**Fig 1:**
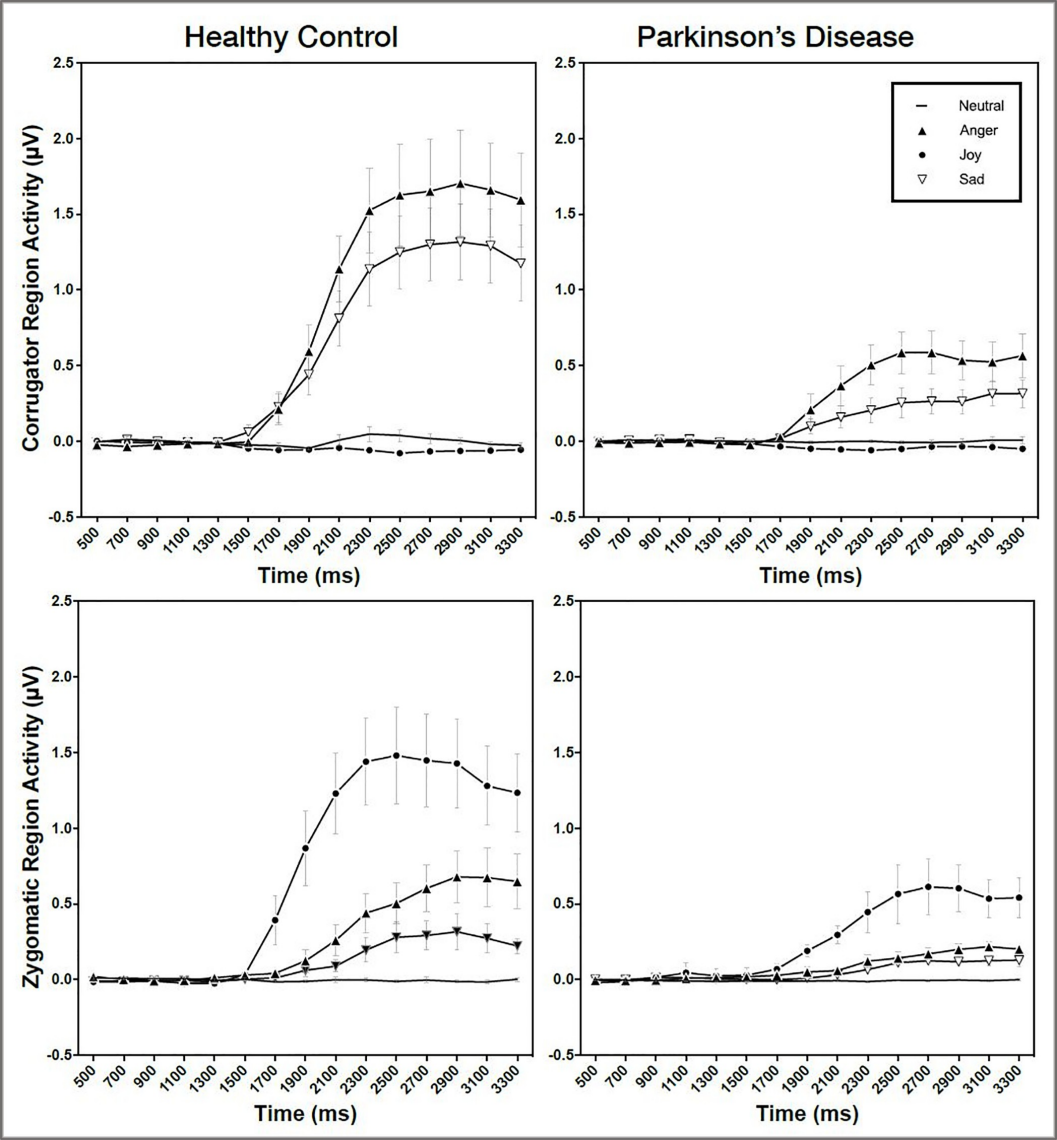
Facial EMG response in the voluntary condition.

Facial EMG responses in the voluntary conditions were demonstrated as shown in Fig 1.

#### Corrugator region response

For the corrugator region, significant main effects of emotion (F (2242) = 235.82, p < .001, f^2^ = .817) and interval (F (14,2242) = 40.53, p < .001, f^2^ = .295) were observed. The interaction between emotion and interval (F(42,2242) = 15.58, p < .001, f^2^ = .148) was also significant. The group difference (F (1,2242) = 13.98, p < .001, f^2^ = .184) was significant, and the interactions between emotion and group (F (42,2242) = 68.06, p < .001 f^2^ = .156), interval and group (F(14,2242) = 12.94, p < .001, f^2^ = .045) were also significant. Finally, the three-way interaction between emotion and interval and group was significant (F(42,2242) = 4.24, p < .001 f^2^ = .017) (Table 1).

**Table 1:** Facial EMG activities in Voluntary and spontaneous mimic conditions.

Effects		Num df	Den DF	Voluntary mimic condition		Spontaneous mimic condition	
				F	P	F	P
Emotion	Cor	3	2242	235.82	< .0001***	28.96	< .0001***
	Zyg	3	2242	192.94	< .0001***	91.59	< .0001***
Emotion x Group	Cor	3	2242	68.06	< .0001***	8.04	< .0001***
	Zyg	3	2242	41.36	< .0001***	20.63	< .0001***
Time	Cor	14	2242	40.53	< .0001***	1.50	0.1019
	Zyg	14	2242	36.39	< .0001***	10.17	< .0001***
Time x Group	Cor	14	2242	12.94	< .0001***	0.96	0.4953
	Zyg	14	2242	8.00	< .0001***	1.67	.0552
Emotion x Time	Cor	42	2242	15.58	< .0001***	1.25	0.1277
	Zyg	42	2242	11.37	< .0001***	4.16	< .0001***
Emotion x Time x Group	Cor	42	2242	4.24	< .0001***	0.57	0.9873
	Zyg	42	2242	2.71	< .0001***	0.82	.7933
group	Cor	1	2242	13.98	.0002***	0.99	0.3188
	Zyg	1	2242	8.24	.0041**	2.08	.1495

Main effects of within-subject factors Emotion (neutral, anger, joy, sad) and time and the between-subjects factor group (patient, healthy control), along with interactions.

Σ of squ., sum of squares.

* < .05

** < .01

*** < .001.

Cor, corrugator region; Zyg, zygomaticus region.

Post-hoc pairwise-comparisons were made to examine the group differences on the muscle region activation known to relate to specific emotions. In comparison to the PD group, the HC group showed greater voluntary corrugator activity for Anger (p < .001) and Sad (p < .001) conditions (Table 2).

**Table 2:** Post hoc pairwise comparison in voluntary and spontaneous mimic condition.

			Voluntary mimic condition			Spontaneous mimic condition		
			Mean	t	Bonferroni-adjusted *P*	Mean	t	Bonferroni-adjusted *P*
HC-PD	Cor	Neutral	-0.00242	-0.03	0.9732	0.01141	0.61	0.5446
		Anger	0.5174	7.19	< .0001***	0.02715	1.44	0.1493
		Joy	-0.01361	-0.19	0.8501	-0.01416	-0.75	0.452
		Sad	0.4734	6.58	< .0001***	0.04431	2.35	0.0187*
	Zyg	Neutral	0.003937	0.06	0.9533	0.001609	0.12	0.9032
		Anger	0.1873	2.78	0.0054**	0.005896	0.45	0.656
		Joy	0.4538	6.75	< .0001***	0.06032	4.56	< .0001***
		Sad	0.06323	0.94	0.3473	0.001683	0.13	0.8988

Σ of squ., sum of squares.

* < .05

** < .01

*** < .001.

Cor, corrugator region; Zyg, zygomaticus region.

Within-group comparisons also revealed the differences between specific emotions. In the control group, post-hoc tests revealed greater corrugator activity toward Angry faces as compared to Neutral (p < .001), joy (p < .001), and Sad (p < .001) faces. Similarly, the corrugator activity was greater toward Sad faces than toward both Neutral (p < .001) and Joy (p < .001) faces. The PD group revealed a similar pattern: greater corrugator activity toward Angry faces compared to Neutral, Joy, and Sad faces, and greater activity for Sad faces compared to Joy and Neutral faces (all p < .001).(Table 3)

**Table 3:** Adjust means and post hoc pairwise comparison in voluntary and spontaneous mimic condition.

			Voluntary Mimic Condition			Spontaneous Mimic Condition		
			Mean	t	Bonferroni-adjusted *P*	Mean	t	Bonferroni-adjusted P
PD	Cor	Anger-Neutral	0.2551	7.23	< .0001	0.01971	2.25	0.0246*
		Anger-Joy	0.2836	8.03	< .0001	0.02447	2.79	0.0053**
		Anger-Sad	0.1289	3.65	0.0003	0.003776	0.43	0.6665
		Joy-Neutral	-0.02851	-0.81	0.4195	-0.004764	-0.54	0.5867
		Joy-Sad	-0.1547	-4.38	< .0001	-0.02069	-2.36	0.0183*
		Sad-Neutral	0.1262	3.57	0.0004	0.01593	1.82	0.0692
	Zyg	Anger-Neutral	0.08751	2.82	0.0049	0.01508	2.39	0.0171*
		Anger-Joy	-0.1816	-5.85	< .0001	-0.02438	-3.86	0.0001***
		Anger-Sad	0.03121	1.01	0.3149	0.001843	0.29	0.7705
		Joy-Neutral	0.2691	8.67	< .0001	0.03946	6.24	< .0001***
		Joy-Sad	0.2128	6.85	< .0001	0.02622	4.15	< .0001***
		Sad-Neutral	0.0563	1.81	0.0699	0.01323	2.09	0.0363
Controls	Cor	Anger-Neutral	0.7749	21.95	< .0001	0.03545	4.05	< .0001***
		Anger-Joy	0.8146	23.07	< .0001	0.06578	7.51	< .0001***
		Anger-Sad	0.1729	4.9	< .0001	-0.01338	-1.53	0.1268
		Joy-Neutral	-0.0397	-1.12	0.2609	-0.03033	-3.46	0.0005
		Joy-Sad	-0.6416	-18.18	< .0001	-0.07916	-9.03	< .0001***
		Sad-Neutral	0.6019	17.05	< .0001	0.04883	5.57	< .0001***
	Zyg	Anger-Neutral	0.2708	8.72	< .0001	0.01936	3.06	0.0022**
		Anger-Joy	-0.4481	-14.43	< .0001	-0.0788	-12.47	< .0001***
		Anger-Sad	0.1552	5	< .0001	0.006056	0.96	0.3379
		Joy-Neutral	0.719	23.16	< .0001	0.09817	15.54	< .0001***
		Joy-Sad	0.6034	19.43	< .0001	0.08486	13.43	< .0001***
		Sad-Neutral	0.1156	3.72	0.0002	0.01331	2.11	0.0353

Σ of squ., sum of squares.

* < .05

** < .01

*** < .001.

Cor, corrugator region; Zyg, zygomaticus region.

#### Zygomaticus region response

For the zygomaticus region, there were significant effects of emotions (F (2242) = 192.94, p < .001, f^2^ = .591), and interval (F(14,2242) = 36.39, p < .001, f^2^ = .215). The interaction between emotion and interval (F (42,2242) = 11.37, p < .001, f^2^ = .087) was also significant. The group difference (F (1,2242) = 8.24, p = .004, f^2^ = .102) was significant, and interactions between emotion and group (F (42,2242) = 41.36, p < .001, f^2^ = .087), and interval and group, (F(14,2242) = 8.00, p < .001, f^2^ = .021) were also significant. The three-way interaction between emotion and interval and group was significant (F(42,2242) = 2.71, p < .001, f^2^ = .006)(Table 1).

Post-hoc pairwise-comparisons were conducted to examine the group difference on the muscle region activation known to be related to specific emotions: in comparison to the PD group, the HC group showed greater degree of voluntary zygomaticus activity toward Anger (p = .005) and Joy (p < .001) faces (Table 2).

Within-group comparisons also revealed differences between specific emotions. In the control group, post-hoc tests revealed greater zygomaticus activity toward Joy faces as compared to Neutral (p < .001), Anger (p < .001), and Sad (p < .001) faces. Angry faces also showed greater activity as compared to Neutral (p < .001), and Sad (p < .001) conditions as did the Sad face compared to the Neutral face (p < .001). This pattern was largely replicated in the PD group: greater corrugator activity toward Joy faces compared to Neutral (p < .001), Anger (p < .001), and Sad (p < .001) faces. In addition, Angry faces showed greater activity compared to Neutral (p = .005), but not to Sad (p = .319) faces. The study found marginally significant differences between Sad and Neutral faces (p = .070) (Table 3).

#### Spontaneous mimicry condition

**Fig 2:**
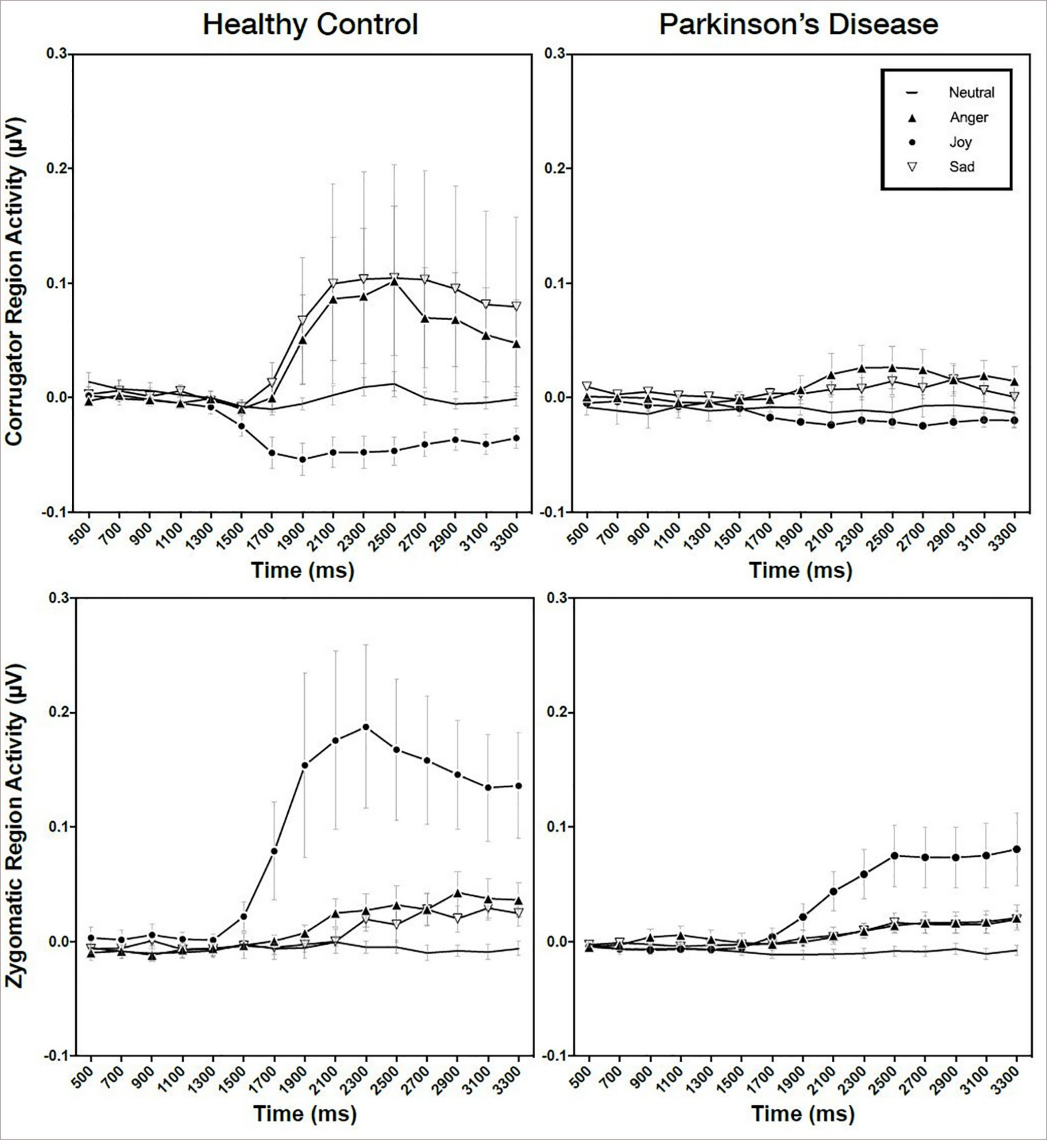
Facial EMG response in the spontaneous condition.

Facial EMG responses in the spontaneous conditions were demonstrated as shown in Fig 2.

#### Corrugator region response

There was a significant main effect of emotion (F (2242) = 28.96, p < .001, f^2^ = .051), but main effects of interval (F(14,2242) = 1.50, p = .102, f^2^ = .021), and the interaction between emotion and interval, (F(42,2242) = 1.25, p < .128, f^2^ = .19), were not significant. The group difference (F (1,2242) = 0.99, p = .319, f^2^ = .009), and the interaction between interval and group (F(14,2242) = 0.96, p = .495, f^2^ = .015), were not significant, but the interaction between emotion was group was significant (F(3,2242) = 8.04, p < .001, f^2^ = .012). The three-way interaction between emotion and interval and group (F(42,2242) = 0.57, p < .987, f^2^ = .012), was not significant (Table 1). In comparison to the PD group, the HC group did show greater spontaneous corrugator activity in the Sad condition (p = .019) (Table 2).

Within-group comparisons revealed that in control group, Angry faces had greater corrugator activity as compared to Neutral (p < .001) and Joy (p < .001), but not toward Sad (p < .127) faces. Similarly, the activity toward Sad faces was greater than toward Neutral (p < .001) and Joy (p < .001) faces. This was replicated in the PD group with greater corrugator activity toward Angry faces as compared to Neutral (p = .025), joy (p = .005) and toward Sad faces as compared to Joy (p = .018) faces with a trend for Neutral faces (p = .069) (Table 3).

#### Zygomaticus region response

Significant main effects of emotion (F (2242) = 91.59, p < .001, f^2^ = .219) and interval (F(14,2242) = 10.17, p < .001, f^2^ = .036) were observed. The interaction between emotion and interval (F(42,2242) = 4.16, p < .001, f^2^ = .010) was also significant. The group difference (F (1,2242) = 2.08, p = .150, f^2^ = .010), and the interaction between interval and group (F(14,2242) = 1.67, p = .055, f^2^ = .011) were not significant, but the interaction between emotion and group (F(3,2242) = 20.63, p < .001, f^2^ = .012) was. The three-way interaction between emotion and interval and group was not significant (F(42,2242) = 0.82, p = .793, f^2^ = .008) (Table 1).

Post-hoc pairwise-comparisons showed that the PD group had greater spontaneous zygomaticus activity in the Joy (p < .001) condition compared to the HC group (Table 2).

Within-group comparisons showed that both controls and PD groups had greater zygomaticus activity toward Joy faces as compare to Neutral (both p < .001), Anger (both p < .001), and Sad (both p < .001) faces. Similarly, both groups had greater activity toward Angry faces than toward Neutral faces (HC: p = .002; PD: p = .017) (Table 3).

#### The relationship between facial muscle activity and self-report information

Correlations between the maximum and mean activity of each muscle regions toward the associated emotions with self-report measures were assessed next [39]. Due to multiple comparisons that were made (two muscle regions and two conditions), we applied a Bonferroni correction that yielded a corrected alpha value of 0.05/4 = 0.0125 for tests.

#### The relationship with subjective measures of well-being

We conducted a partial correlation analysis for age and sex, which revealed significant correlations between the patients’ well-being score and facial muscle activation. Specifically, in the spontaneous condition, mean EMG activation of the zygomatic region in the resting condition correlated with the patients’ well-being score (r = .471, p = .003). Among the subscales, emotional well-being (r = .549 p < .001) and social well-being (r = .464, p = .003) were significant. The correlation with the psychological well-being subscale was not significant according to the Bonferroni-corrected analysis (r = .360, p = .026). Similarly, the maximum EMG activation of the zygomatic region also had significant correlations with the patients’ well-being score (r = .479, p = .002). Among the subscales, emotional well-being (r = .552, p < .001) and social well-being (r = .438, p = .006) were significant, except for the psychological well-being subscale (r = .360, p = .026).

In the voluntary mimicry condition, the mean and maximum zygomatic activation toward Joy faces subtracted from the Neutral condition correlated with the social well-being subscale at a marginal level (mean: r = .328 p = .045; max: r = .351, p = .030).

Relationships for corrugator activity and negative emotions (Anger, Sad) were not significant in both voluntary and spontaneous conditions.

Finally, there were significant correlations between the activation of zygomatic region toward Angry faces in the voluntary, but not in the spontaneous condition: the mean activation correlated with the total subjective well-being score (r = .356, p = .028), and the social well-being subscore (r = .430, p = .007), and the maximum activation with the total patients’ well-being score (r = .378, p = .019), the social well-being subscore (r = .459; p = .004), and the psychological well-being subscore (r = .330, p = .043).

#### The relationship with clinical indices of PD symptoms

There was no significant relationship between facial EMG activity and clinical indices. The partial correlations between UPDRS part III score and the indices of facial EMG were not significant (p = .103~.987) as were the partial correlations between PDQ score and the indices (p = .027~.964). The maximum activation of corrugator activity toward Sad faces subtracted from the activation toward Neutral ones in the spontaneous condition showed only marginal significance (p = .027, r = -.519).

#### The relationship with alexithymia symptoms

As a whole group, the correlation between facial EMG activity and alexithymia score was not significant (p = .162~.952).

## Discussion

The current study showed that facial mimicry toward dynamic emotional expressions is reduced among individuals with PD. In accordance with previous work [26], the relationship between the observed decrease in facial mimicry and the symptom severity measured with UPDRS-III is not significant. In contrast, reduced facial mimicry is correlated to several indices of subjective quality of life, such as emotional, and social well-being, but not to a widely-used health-related quality of life measurement (PDQ).

Using carefully controlled, dynamic facial expression stimuli, this study showed that facial mimicry of PD patients is reduced in both voluntary and spontaneous conditions. Although characteristic mimicry patterns to each emotion were observed in both control and PD groups, the intensity of the muscle activation in the PD group was significantly smaller compared to that of controls. This finding is consistent with recent studies reporting reduced speed and intensity in voluntary mimicry [26], and spontaneous mimicry [16]. Taken together, the findings echoe Rinn’s original conclusion that individuals with PD are not totally paralyzed, but that they have difficulty in motor expression [40].

Conventionally, the degeneration of nigrostriatal dopaminergic neurons in PD is known to be related to the alteration of the basal ganglia thalamocortical networks which in turn causes motor symptoms. In line with this view, recent imaging studies reported hypo-activation of supplementary motor area in PD [41–43]. The alteration of motor cortex function seems to play an essential role in reduced facial mimicry commonly observed in both voluntary and spontaneous conditions [44, 45] as also evidenced by results with transcranial magnetic stimulation showing that M1 corticobulbar projections elicit MEPs in facial muscles [45]. Specifically, there are several suggested pathways for voluntary and spontaneous facial expressions [46, 47]. Results from early case studies established that an intact motor cortex is required for voluntary facial muscle movement, whereas spontaneous movements can be generated with basal ganglia and extrapyramidal system functions [48, 49]. More recent fMRI studies have reported primary motor cortex and supplementary motor cortex activation along with right frontal areas during voluntary imitation of facial expressions [50]. Studies of spontaneous mimicry also report activation of primary and supplementary motor cortices [51]. Furthermore, results from a recent neuromodulation study with rTMS stimulation over the primary motor cortex also point to reduced spontaneous facial mimicry and hence to an involvement of the primary motor cortex [52].

The observed impairment in spontaneous facial expressions in PD in this study might not be due to a lesion of the motor cortex, but rather due to basal ganglia degeneration which is crucial for the fluidity and spontaneity of the movement, since the loss of fluidity and spontaneity not only directly affects spontaneous facial expression, but also impairs the fluidity of voluntary expressions [40]. This result is consistent with with a recent deep brain stimulation study in which subthalamic stimulation increased both voluntary and spontaneous blinking [53].

Emerging evidence suggests that amygdala dysfunction also accompanies the degeneration of substantia nigra in PD [54] with concurrent pathological changes in central and accessory cortical nuclei of amygdala as well as substantia nigra in this population. Since the amygdala is a hub of emotional processing in the brain [55], one may suppose that amygdala dysfunctions can also alter facial expression generation. Evidence for this connection, however, is equivocal: amygdala lesions leads to increased brain activation in emotion processing elsewhere in the brain [56], but does not impair the voluntary generation of facial expressions [57]. Similarly, a recent review pointed out that although there is a general hypofunction of the amygdala in PD medication induces a hyperfunction [54]. The current study examined PD patients in the off-drug state therefore preventing potential effects from drug-induced amygdala hyperfunction.

Interestingly, the present analysis did not find any relationship between decreased facial mimicry and the disease-related quality of life measured via PDQ. Instead, a significant relationship was observed between the extent of patient’s facial mimicry and subjective well-being indices. The result indicates that the ‘masked face’ symptom affects more social and psychological aspects of the patient’s quality of life. Impaired facial expression production is known to directly degrade quality of life [58] and indirectly makes patients’ social life more challenging [59, 60]. Since both voluntary and spontaneous mimicry play important roles in emotional communication, including understanding[1], sharing others’ emotion [2, 61] and social affiliation [8, 11], the decrease of facial mimicry might further impair emotional communication. Along with previous studies [24, 62], the study did not find any relationship between the decrease and alexithymia. This suggests that the impaired facial mimicry is not a symptom of alexithymia or comorbid depression, which frequently occur in PD [58, 63], but rather that it is one of the possible precursors of depressive symptoms, via lowering quality of life in a social context.

## Conclusion

Together, the findings of current study provide clear evidence for the impairment of facial mimicry in PD. The facial EMG analysis showed that these impairments exist in both voluntary and spontaneous mimicry. This impairment seems not related to alexithymia, nor is there a relation with a widely-used healthy related quality of life measurement (PDQ). Instead, impaired mimicry correlates with quality of life measurements reflecting more subjective, psychological aspects (SWB).

### Limitation

This study had the following limitations: first, although it adopted dynamic facial expressions with more ecological validity compared to static ones, the expressions that were used were those of younger people, due to the lack of a suitable database of elderly faces. A possible effect of the age of the stimulus faces should be considered in future studies [64]. Second, although all participants withdrew from using their dopaminergic medications for at least 12 hours, the medication might either way have affected the results of this study. A further study using a longitudinal design with repeated sEMG measurements following the disease progression is needed to confirm the result in the context of the pathophysiology and to elucidate the causal relationship between non-motor features of social well-being, emotional feelings and motor features of facial expressivity in PD.

## Supporting information

S1 File (CSV)
