# Correction: Voluntary and spontaneous facial mimicry toward other’s emotional expression in patients with Parkinson’s disease

**DOI:** 10.1371/journal.pone.0217715

**Published:** 2019-05-28

**Authors:** June Kang, Dilara Derva, Do-Young Kwon, Christian Wallraven

In the Stimulus subsection of the Materials and methods section, there is an incorrect reference in the third sentence.

The correct sentence is: Expression stimuli were chosen from the East-Asian Dynamic Facial Expression Stimulus (EADFES) database (Kang et al., 2015), which contains stimuli created and verified based on suggested action units related to each emotion (Anger: 4CDE+5CED+7+17+23/24, Joy: 6+12CDE+25, Sad: 1+4+15ABC+17, [38]).

The reference is: Kang J, Ham BJ. Development and Validation of East-Asian Dynamic Facial Expression Stimulus. Proceedings of Current Trends in Biomedical Science. 2015. Seoul, South Korea.
